# Severe weight loss during preoperative chemoradiotherapy compromises survival outcome for patients with locally advanced rectal cancer

**DOI:** 10.1007/s00432-016-2225-1

**Published:** 2016-09-09

**Authors:** Junzhong Lin, Jianhong Peng, Aiham Qdaisat, Liren Li, Gong Chen, Zhenhai Lu, Xiaojun Wu, Yuanhong Gao, Zhifan Zeng, Peirong Ding, Zhizhong Pan

**Affiliations:** 1 Department of Colorectal Surgery, Sun Yat-sen University Cancer Center, State Key Laboratory of Oncology in South China, Collaborative Innovation Center for Cancer Medicine, 651 Dongfeng Road East, Guangzhou, 510060 People’s Republic of China; 2 Department of Emergency Medicine, The University of Texas MD Anderson Cancer Center, Houston, TX 77030 USA; 3 Department of Radiation Oncology, Sun Yat-sen University Cancer Center, State Key Laboratory of Oncology in South China, Collaborative Innovation Center for Cancer Medicine, Guangzhou, 510060 People’s Republic of China

**Keywords:** Weight loss, Preoperative chemoradiotherapy, Survival outcome, Rectal cancer

## Abstract

**Purpose:**

In addition to tumor factors, poor nutritional status before and during anti-tumor treatment might compromise prognosis in several types of cancer. This study was done to determine the impact of weight loss during preoperative chemoradiotherapy (CRT) on the survival outcome of patients with locally advanced rectal cancer (LARC).

**Methods:**

The retrospective study examined consecutive patients with LARC who underwent preoperative CRT followed by radical resection in a single institute, between 2003 and 2013. Correlation of proportional body mass index (BMI) change after preoperative CRT and patient’s demographics, tumor characteristics, treatment parameters, CRT-related toxicity, disease-free survival (DFS) and overall survival (OS) were investigated.

**Results:**

A total of 364 patients were enrolled, and BMI loss was found in 243 patients (66.2 %) after preoperative CRT. Severe weight loss (SWL) was defined as BMI loss ≥7 %. Thirty-nine (10.7 %) cases were enrolled in SWL cohort and found to have higher incidence of diarrhea (*P* = 0.033), renal disorder (*P* = 0.033) and grade 3–4 radiation proctitis (*P* = 0.041). Although no significant difference was found in 3-year DFS, patients in SWL cohort showed significantly worse 3-year OS rate (71.8 vs 88.0 %, *P* = 0.030) than the others. In univariate analysis, BMI loss ≥7 %, completed dose of preoperative chemotherapy, pathologic T and N stages were correlated with OS (all *P* < 0.05). In multivariable analysis, BMI loss ≥7 % (HR 1.984; 95 % CI 1.061–3.709; *P* = 0.032) remained the independent prognostic factor for OS.

**Conclusions:**

Our results demonstrate that SWL during preoperative CRT indeed compromises survival outcome in patients with LARC. Routine nutritional monitoring and nutritional support during preoperative CRT are suggested as the integral part of the multidisciplinary approach for these patients.

## Introduction

Preoperative chemoradiotherapy (CRT) followed by total mesorectal excision (TME) has been demonstrated as the effective treatment pattern for patients with locally advanced rectal cancer (LARC) (Bosset et al. 2006; Sauer et al. 2004). Although most of these patients could achieve improvement of local disease control and sphincter preservation, a proportion of patients failed to benefit from preoperative CRT (Diaz-Gonzalez et al. 2006; Kong et al. 2012). Therefore, it is necessary to identify risk factors associated with prognosis and individualize the therapy based on the oncologic outcome analysis. In addition to tumor factors, many studies supported the idea that poor nutritional status before and during anti-tumor treatment could compromise prognosis in some types of cancer (Cooper et al. 2015; Iseki et al. 2015; Sakurai et al. 2016).

Body mass index (BMI), as a crucial indicator in the assessment of nutritional status, is easy to measure with low cost and may be associated with mortality of patients suffering from cancer (Parr et al. 2010; Reeves et al. 2007). Recently, lower preoperative BMI has been identified as a risk factor for poor prognosis of rectal cancer (Adachi et al. 2015; Uratani et al. 2015). Since malnutrition commonly occurred during chemotherapy and radiotherapy, the changes in nutritional status during treatment should be taken into consideration (Gudny Geirsdottir and Thorsdottir 2008; Koom et al. 2012). To date, data remain limited about the impact of weight loss during neoadjuvant treatment on long-term survival for LARC patients. We hypothesized that severe weight loss during preoperative CRT resulted in a worse patient’s survival. Herein, we conducted this retrospective study to investigate the association of weight loss during preoperative CRT and the survival outcome in patients with LARC.

## Patients and methods

### Patient selection

Patients with LARC undergoing preoperative CRT followed by total mesorectal excision at Sun Yat-sen University Cancer Center during March 2003–April 2013 were retrospectively identified. Enrolled patients met the following inclusion criteria: (1) histologically confirmed rectal adenocarcinoma; (2) T3–4 or N+ disease initially; and (3) radical resection for rectal tumor. The exclusion criteria were as follow: (1) missing height and weight records of pre- or post-CRT; (2) metastatic disease before or during preoperative treatment; and (3) other active malignancy (except for basal cell carcinoma of the skin). Patient demographic, preoperative and postoperative treatment, CRT toxic reaction and follow-up results were reviewed in detail from the medical records and the follow-up system. Current study was undertaken in accordance with the ethical standards of the World Medical Association Declaration of Helsinki. A waiver of informed consent was requested, and the approval was obtained from independent ethics committees at Sun Yat-Sen University Cancer Center.

### Treatments

All patients underwent preoperative CRT in the parallel pattern. Irradiation was scheduled in total dose of 46.0–50.40 Gy to the pelvic area, delivered in fractions of 1.8 or 2.0 Gy daily on five consecutive days per week during 6 weeks. Concurrent chemotherapy regimen was administered as follow: XELOX regimen (oxaliplatin 130 mg/m^2^ on day 1 and capecitabine 1000 mg/m^2^ twice daily on days 1–14 were given for 3 week-cycle); or FOLFOX6 regimen (oxaliplatin 85 mg/m^2^ and leucovorin 400 mg/m2 were administered on day 1, 5-FU was injected intravenously 400 mg/m^2^ on day 1 and then administered 2400 mg/m^2^ by intravenous infusion on days 1 and 2 for 2 week-cycle); or capecitabine only (capecitabine 825 mg/m^2^ was given twice daily during radiotherapy without weekend breaks). All patients underwent radical TME resection 6–8 weeks after the end of the preoperative radiotherapy. The recommended oxaliplatin-based adjuvant chemotherapy started 3–6 weeks after surgery.

### Measurements

Height and weight of overall patients were measured 1 day before CRT and surgery. BMI was calculated as weight (kg)/height square (m^2^), and its relative change was generated. BMI level was defined according to the Chinese standard (Zeng et al. 2014): higher BMI (overweight or obese), >24 kg/m^2^; normal BMI, 18.5–24 kg/m^2^; and lower BMI (underweight), ≤18.5 kg/m^2^. Pretreatment staging was determined by endorectal ultrasound (EUS), chest and abdominopelvic computed tomography scanning (CT) and/or pelvic magnetic resonance imaging (MRI). Acute toxicities during CRT were graded according to the National Cancer Institute Common Toxicity Criteria (NCI CTC) version 4.0. Pathological assessments of the resected specimens were confirmed according to tumor-node-metastasis (TNM) classification by two independent pathologists. Pathologic complete response (pCR) was defined as follow: the absence of viable tumor cells with only fibrotic masses or a cellular mucin pools present in area of primary tumor and lymph nodes (Mandard et al. 1994).

### Follow-up

The primary endpoint was overall survival (OS), and the secondary endpoints were disease-free survival (DFS). OS was defined as time length from operation to death from any cause, while DFS was defined as the interval from tumor resection to disease recurrence. All patients were observed through subsequent visit every 3 months for 2 years and then semiannually until 3 years after surgery. Evaluation included clinical examination, carcinoembryonic antigen (CEA) level, abdominal ultrasonography and chest radiograph. A chest computed tomography (CT), abdominal/pelvic magnetic resonance (MRI) and colonoscopy were performed annually. The last follow-up visit occurred in March 2016.

### Statistical analysis

Clinical data were analyzed using Statistical Package for the Social Sciences (SPSS, version 17.0, Chicago, IL). Continuous variables were presented as mean (standard deviation, SD) or median (range), which were compared using Student *t* test or Mann–Whitney test. Categorical variables were given as percentage and then compared by applying Chi-square test or Fisher’s exact between groups, when appropriate. Receiver operating characteristic curve (ROC) analysis was used to determine the cutoff point of BMI change according to OS. The OS and DFS rates were estimated by the Kaplan–Meier method, and differences between the two groups were assessed by the log-rank test. Variables proved statistical significance in univariate Cox models were further assessed by multivariate Cox models. The multivariate Cox proportional hazards model was used to identify independent prognostic factors for OS, and the hazard ratios (HRs) and confidence intervals (CIs) were calculated. All statistical tests were two-sided, and the significant level was set at 0.05.

## Results

### Patient characteristics and cutoff point for weight loss

A total of 364 patients were enrolled. The characteristics of BMI and body weight for these patients are shown in Table 1. Initially, 40 (11 %) patients were underweight, while 108 (29.7 %) were overweight. BMI and weight significantly decreased during CRT, especially for overweight patients, and BMI loss was found in 243 patients (66.2 %).

**Table 1:** Body mass index and body weight change during preoperative chemoradiotherapy with patients of locally advanced rectal cancer

Factors	*N* (%)	Pre-CRT	Post-CRT	Relative change (median, range)	Variation (95 % CI)	*P* value
Pre-CRT BMI (mean ± SD, kg/m^2^)						
Total	364	22.4 ± 3.1	22.3 ± 3.1	−0.71 % (−25.97 to 30.91 %)	−0.163 (−0.295 to −0.030)	0.02
≤18.5	40 (11.0)	17.2 ± 1.3	17.4 ± 1.6	0 (−9.96 to 22.08 %)	0.257 (−0.143 to 0.657)	0.20
18.5–24	216 (59.3)	21.6 ± 1.5	21.5 ± 1.8	0 (−14.29 to 30.91 %)	−0.077 (−0.240 to 0.086)	0.35
>24	108 (29.7)	26.1 ± 1.7	25.6 ± 2.0	−1.29 % (−12.70 to 25.97 %)	−0.489 (−0.754 to −0.225)	<0.001
PreCRT body weight (mean ± SD, kg)						
Total	364	60.5 ± 9.7	60.1 ± 9.8	−0.71 % (−25.97 to 30.91 %)	−0.418 (−0.771 to −0.056)	0.02
≤50	50 (13.7)	45.5 ± 4.1	45.7 ± 5.1	−1.10 % (−11.46 to 22.08 %)	0.224 (−0.724 to 1.172)	0.64
50–70	256 (70.3)	60.1 ± 5.7	59.7 ± 6.2	0 (−17.07 to 30.91 %)	−0.330 (−0.731 to 0.071)	0.11
>70	58 (15.9)	75.4 ± 4.2	74.1 ± 5.8	−1.32 % (−25.97 to 8.97 %)	−1.333 (−2.458 to −0.207)	0.02

*BMI* body mass index, *SD* standard deviation, *CI* confidence interval, *CRT* chemoradiotherapy

**Fig. 1:**
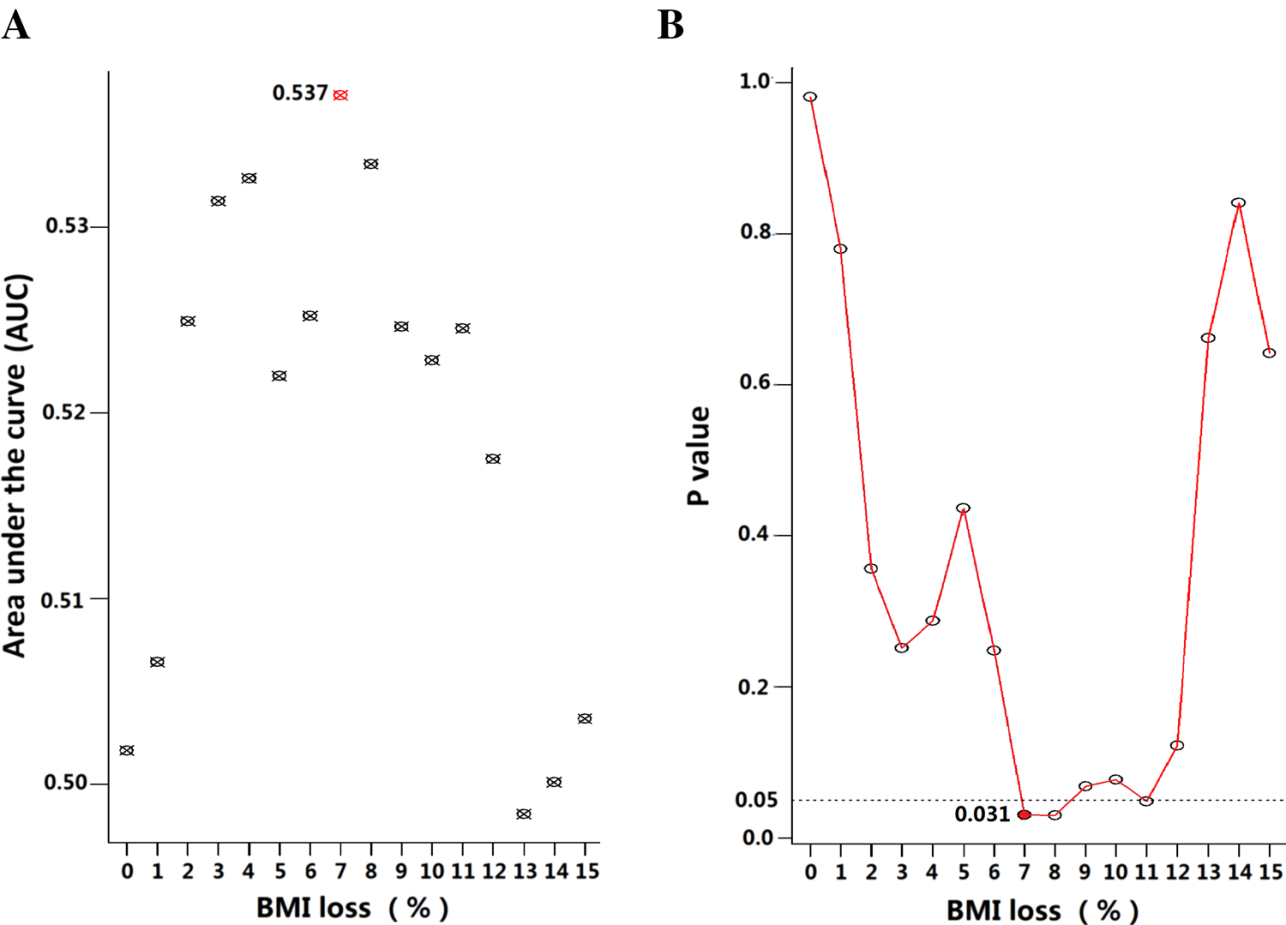
Comparison of different cutoff points of BMI loss based on overall survival outcome by **a** area under the curve (AUC) and **b** the minimal *P* value

The receiver operating characteristic (ROC) analysis indicated the optimal cutoff value of BMI change for OS was 7 % (*P* = 0.031, Fig. 1). Therefore, severe weight loss (SWL) during CRT was defined as BMI loss ≥7 %. As a result, 39 (10.7 %) patients with BMI loss ≥7 % were included in the SWL cohort, while the others with BMI loss <7 %, no BMI loss or even BMI gain during CRT were grouped into non-severe weight loss (non-SWL) cohort.

Patients in SWL cohort seemed to be older than those in non-SWL cohort (62 vs 55 years, *P* = 0.007). Despite of age, clinical characteristics of the two groups including gender distribution, baseline BMI, distance of inferior tumor margin from the anal verge (DAV), tumor size and pretreatment TNM stage were comparable (Table 2). In addition, there was no significant difference in the pathological outcome after CRT and surgical resection between the two groups.

**Table 2:** Clinical and pathological characteristics of patients with locally advanced rectal cancer

Variables	All patients *n* = 364 (%)	BMI loss <7 % *n* = 325 (%)	BMI loss ≥7 % *n* = 39 (%)	*P* value
*Clinical variables*				
Age, median (range)	56 (15–80)	55 (15–80)	62 (33–76)	0.007
Gender (male: female)	259:115	224:101	25:14	0.54
Mean preCRT BMI (kg/m^2^)	22.5 ± 3.1	22.4 ± 3.1	22.6 ± 2.9	0.72
Mean inferior tumor margin from anal verge, cm (SD)	4.9 ± 2.2	4.9 ± 2.1	5.2 ± 2.7	0.35
cT stage				0.46
3	216 (59.3)	195 (60.0)	21 (53.8)	
4	148 (40.7)	130 (40.0)	18 (46.2)	
cTNM stage				0.12
II	127 (34.9)	109 (33.5)	18 (46.2)	
III	237 (65.1)	216 (66.5)	21 (53.8)	
*Pathological variables*				
Mean size of tumor after CRT, cm (SD)	3.0 ± 1.6	3.0 ± 1.5	3.4 ± 1.9	0.14
Tumor differentiation				0.90
Well and moderate	283 (77.7)	253 (77.8)	30 (76.9)	
Poor	81 (22.3)	72 (22.2)	9 (23.1)	
Median number of lymph nodes examined (range)	6 (0–37)	6 (0–37)	6 (0–21)	0.99
Median number of positive lymph nodes (range)	0 (0–12)	0 (0–12)	0 (0–4)	0.42
ypT stage				0.42
0–2	181 (49.7)	164 (50.5)	17 (43.6)	
3–4	183 (50.3)	161 (49.5)	22 (56.4)	
ypN stage				0.50
0	283 (77.7)	251 (77.2)	32 (82.1)	
1–2	81 (22.3)	74 (22.8)	7 (17.9)	
ypTNM stage				0.87
0	85 (23.4)	76 (23.4)	9 (23.1)	
I	77 (21.2)	69 (21.2)	8 (20.5)	
II	121 (33.2)	106 (32.6)	15 (38.5)	
III	81 (22.2)	74 (22.8)	7 (17.9)	
Achievement of pCR				0.97
Yes	85 (23.4)	76 (23.4)	9 (23.1)	
No	279 (76.6)	249 (76.6)	30 (76.9)	

*BMI* body mass index, *SD* standard deviation, *cTNM stage* clinical tumor-node-metastasis classification, *cT stage* clinical tumor stage, *ypT stage* pathologic tumor stage after chemoradiotherapy, *ypN stage* pathologic node stage after chemoradiotherapy, *ypTNM stage* pathologic tumor-node-metastasis classification after chemoradiotherapy, *pCR* pathologic complete response

### Treatment characteristics and chemoradiotherapy toxicity

In regard to treatment parameters of preoperative CRT and surgery, no significant difference was found between SWL and non-SWL cohorts, as presented in Table 3. However, a smaller proportion in SWL cohort received postoperative chemotherapy (69.2 vs 83.4 %, *P* = 0.030), and a shorter duration of chemotherapy was delivered (10.5 vs 12 weeks, *P* = 0.019), compared to that in non-SWL cohort.

**Table 3:** Treatment characteristics for patients with locally advanced rectal cancer

Variables	BMI loss <7 % *n* = 325 (%)	BMI loss ≥7 % *n* = 39 (%)	*P* value
*Preoperative chemoradiotherapy*			
Chemotherapy regimen			0.25
XELOX	273 (84.0)	32 (82.1)	
FOLFOX	38 (11.7)	7 (17.9)	
Capecitabine	14 (4.3)	0	
Duration of chemotherapy, cycles (median, range)	2 (1–4)	2 (1–4)	0.27
Completion of chemotherapy	258 (79.4)	28 (71.8)	0.26
Completed dose of chemotherapy >50 %	298 (91.7)	37 (94.9)	0.49
Dose of radiotherapy, Gy (range)	46 (30–50)	46 (30–50)	0.32
Completion of radiotherapy	317 (97.5)	37 (94.9)	0.35
*Surgical operation*			
Interval from the completion of radiation to surgery, weeks (range)	6.8 (1–12)	7 (4–11)	0.99
Surgical procedure			0.66
Anterior resection	187 (57.5)	21 (53.8)	
Abdominal perineal resection	138 (42.5)	18 (46.2)	
*Postoperative treatment*			
Adjuvant chemotherapy			0.030
Yes	271 (83.4)	27 (69.2)	
No	54 (16.6)	12 (30.8)	
Duration of postoperative chemotherapy, weeks (median, range)	12 (0–18)	10.5 (0–18)	0.019

*BMI* body mass index, *CRT* chemoradiotherapy

Treatment-related adverse events during preoperative CRT are shown in Table 4. Despite similar incidence in the majority of the adverse events, patients with BMI loss ≥7 % had significantly higher occurrence rate of diarrhea (48.7 vs 31.4 %, *P* = 0.033), renal disorder (5.1 vs 0.6 %, *P* = 0.033) and severe (grade 3–4) radiation proctitis (7.7 vs 1.8 %, *P* = 0.041).

**Table 4:** Treatment-related adverse events during preoperative chemoradiotherapy in patients with locally advanced rectal cancer

Toxicity	BMI loss <7 % *n* = 235 (%)	BMI loss ≥7 % *n* = 39 (%)	*P* value	Toxicity	BMI loss <7 % *n* = 235 (%)	BMI loss ≥7 % *n* = 39 (%)	*P* value
*Leucopenia*				*Rash*			
Total	190 (58.5)	26 (66.7)	0.33	Total	40 (12.3)	3 (7.7)	0.40
Grade 1–2	176 (54.2)	25 (64.1)	0.24	Grade 1–2	36 (11.1)	1 (2.6)	0.13
Grade 3–4	14 (4.3)	1 (2.6)	0.61	Grade 3–4	4 (1.2)	2 (5.1)	0.10
*Thrombocytopenia*				*Urination disorder*			
Total	72 (22.2)	9 (23.1)	0.90	Total	10 (3.1)	1 (2.6)	0.86
Grade 1–2	62 (19.1)	9 (23.1)	0.55	Grade 1–2	10 (3.1)	1 (2.6)	0.86
Grade 3–4	10 (3.1)	0	1	Grade 3–4	0	0	
*Nausea/vomiting*				*Renal disorder*			
Total	150 (46.2)	19 (48.7)	0.76	Total	2 (0.6)	2 (5.1)	0.033
Grade 1–2	149 (45.8)	19 (48.7)	0.73	Grade 1–2	2 (0.6)	2 (5.1)	0.033
Grade 3–4	1 (0.3)	0	1	Grade 3–4	0	0	
*Diarrhea*				*Hepatic disorder*			
Total	102 (31.4)	19 (48.7)	0.033	Total	44 (13.5)	7 (17.9)	0.46
Grade 1–2	91 (28.0)	16 (41.0)	0.09	Grade 1–2	42 (12.9)	7 (17.9)	0.39
Grade 3–4	11 (3.4)	3 (7.7)	0.20	Grade 3–4	2 (0.6)	0	1
*Hand*-*foot syndrome*				*Neurotoxicity*			
Total	225 (69.2)	27 (69.2)	1	Total	53 (16.3)	5 (12.8)	0.58
Grade 1–2	216 (66.5)	25 (64.1)	0.77	Grade 1–2	53 (16.3)	5 (12.8)	0.58
Grade 3–4	9 (2.8)	2 (5.1)	0.42	Grade 3–4	0	0	
*Radiation proctitis*							
Total	198 (60.9)	24 (61.5)	0.94				
Grade 1–2	192 (59.1)	21 (53.8)	0.53				
Grade 3–4	6 (1.8)	3 (7.7)	0.041				

*BMI* body mass index

### Survival outcomes and severe weight loss

**Fig. 2:**
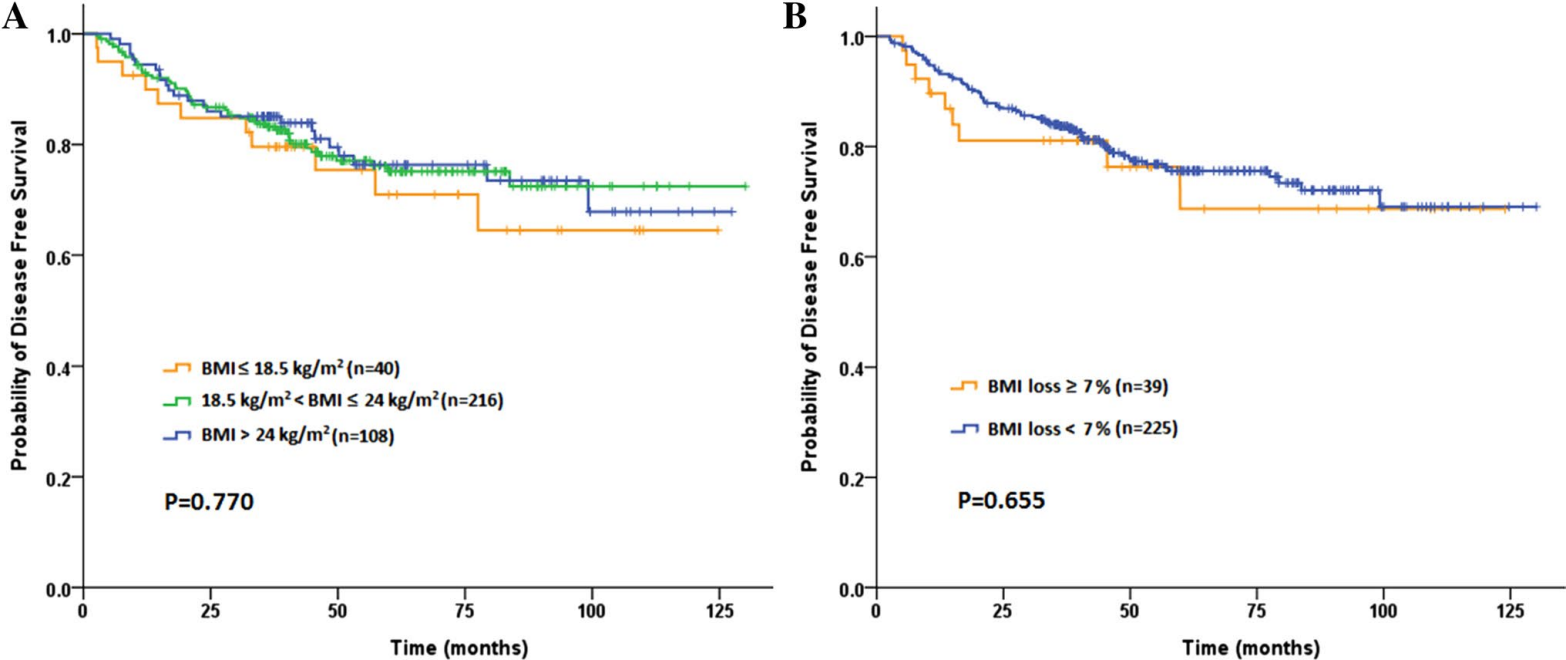
Kaplan–Meier curve comparing 3-year disease-free survival (DFS) rate by **a** baseline BMI classification, **b** BMI loss during preoperative chemoradiotherapy inpatients with locally advanced rectal cancer

**Fig. 3:**
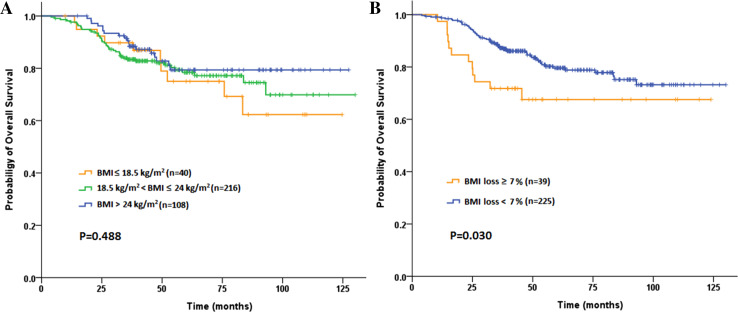
Kaplan–Meier curve comparing 3-year overall survival (OS) rate by **a** baseline BMI classification, **b** BMI loss during preoperative chemoradiotherapy in patients with locally advanced rectal cancer

A total of 83 (22.8 %) patients ultimately developed postoperative recurrences during a median 47.8-month follow-up (range 4–130 months). The 3-year DFS and OS rate were 83.3 and 86.0 %, respectively. Among the baseline subgroups of underweight, normal BMI and overweight or obese patients, neither 3-year DFS rates (79.6 vs 83.7 vs 85.1 %, *P* = 0.770; Fig. 2a) nor 3-year OS rates (89.7 vs 83.4 vs 90.5 %, *P* = 0.488; Fig. 3a), showed any statistical difference. Similarly, no significant difference was found according to the post-CRT BMI (DFS: *P* = 0.820 and OS: *P* = 0.305). Although no significant difference was found in the 3-year DFS (81.1 vs 83.7 %, *P* = 0.655; Fig. 2b), patients in SWL cohort had significantly worse 3-year OS compared to non-SWL cohort (71.8 vs 88.0  %, *P* = 0.030; Fig. 3b). As shown in Table 5, univariate analysis revealed that BMI loss ≥7 % during preoperative CRT (1.961; 95 % CI 1.055–3.647; *P* = 0.030), completed dose of preoperative chemotherapy ≤50 % and advanced pathologic T and N stages were associated with poor OS. The multivariate analysis subsequently demonstrated that all factors above including BMI loss ≥7 % (HR 1.984; 95 % CI 1.061–3.709; *P* = 0.032) were independent prognostic factors for OS.

**Table 5:** Univariate and multivariate analysis of risk factors for overall survival in the patients with locally advanced rectal cancer

Variable	Univariate		Multivariate	
	HR (95 % CI)	*P* value	HR (95 % CI)	*P* value
Age >60 year	1.360 (0.856–2.162)	0.19		
Gender (female)	1.139 (0.702–1.847)	0.60		
PreCRT BMI ≤18.5 kg/m^2^	1.248 (0.640–2.436)	0.52		
PreCRT weight ≤50 kg	1.430 (0.784–2.607)	0.24		
cT stage 4	1.439 (0.867–2.387)	0.16		
cTNM stage III	1.269 (0.734–2.193)	0.39		
DAV ≤ 5 cm	1.158 (0.947–1.417)	0.15		
Tumor size ≥3 cm	1.158 (0.701–1.915)	0.57		
Histopathology (poor grade)	1.175 (0.689–2.002)	0.55		
Number of retrieved LNs ≥12	0.776 (0.456–1.322)	0.35		
ypT stage 3–4	2.411 (1.462–3.976)	0.001	1.781 (1.054–3.010)	0.031
ypN stage 1–2	3.227 (2.031–5.126)	<0.001	2.571 (1.576–4.192)	<0.001
Preoperative CRT with oxaliplatin	0.466 (0.202–1.075)	0.07		
Completed dose of preoperative chemotherapy >50 %	0.470 (0.253–0.874)	0.017	0.532 (0.284–0.997)	0.049
Radiotherapy completion	0.627 (0.195–2.015)	0.43		
Anterior resection	0.694 (0.438–1.100)	0.12		
Acceptance of postoperative chemotherapy	0.798 (0.457–1.393)	0.43		
Duration of postoperative chemotherapy ≥12 weeks	0.843 (0.489–1.453)	0.45		
BMI loss ≥7 % during preoperative CRT	1.961 (1.055–3.647)	0.030	1.984 (1.061–3.709)	0.032

*BMI* body mass index, *HR* hazard ratio, *CI* confidence interval, *DAV* distance of inferior tumor margin from the anal verge, *cT stage* clinical tumor stage, *cTNM stage* clinical tumor-node-metastasis classification, *LNs* lymphonodus, *ypT stage* pathologic tumor stage after chemoradiotherapy, *ypN stage* pathologic node stage after chemoradiotherapy, *CRT* chemoradiotherapy

## Discussion

It is now clear that cancer survival is determined not only by tumor pathology but also by host-related factors, in particular, nutritional status and systemic inflammation (Bachmann et al. 2015; Read et al. 2006). Previous studies have addressed the impact of pretreatment BMI on rectal cancer patient’s outcomes (Campbell et al. 2012; Hede et al. 2015). Moreover, it has been reported that severe weight loss after treatment increased the risk of cancer recurrence and mortality in endometrial cancer and triple-negative breast cancer (Bao et al. 2016; Matsuo et al. 2016). However, the correlation of weight loss during preoperative CRT and survival outcome of LARC patients is not yet established. In this study, we found that severe weight loss during preoperative CRT was the independent risk factor for 3-year OS in LARC patients, instead of the baseline or post-CRT BMI.

Several reasons could contribute to the association between weight loss and poor survival outcome. Of these, the most critical reason is the impact of nutrient on cancer development. Polyunsaturated fatty acids (PUFAs), as one series of essential fatty acids, have been confirmed of having anti-tumor effects by inducing tumor cell apoptosis and inhibiting cell proliferation (Song et al. 2011; Xu and Qian 2014). Weight loss during preoperative CRT is always accompanied by insufficient fatty acids intake, which impairs the therapy effects. On the other hand, the role of immuonutrition is recognized as an important factor in modulating cancer progression. Mounting evidence showed that certain dietary nutrients were associated with the development of different types of cancers either by enhancing the immune system or by exerting an immunosuppressive effect (Valdes-Ramos and Benitez-Arciniega 2007; Yaqoob and Calder 2007). Previous study revealed that once weight loss occurs during treatment, immune function might be impaired, especially cell-mediated immunity (Fontana et al. 2007). Another crucial factor for decreased survival outcome in patients with SWL during CRT is possibly the different compliance of postoperative chemotherapy. In our study, a significant lower proportion of patients in SWL cohort received postoperative chemotherapy, and these patients even had a shorter duration of chemotherapy, suggesting that the malnutrition status during CRT could compromise postoperative treatment compliance, which might influence the survival outcome.

As for the cause of weight loss, CRT-related toxicity may account for that. Our study showed that patients with BMI loss ≥7 % experienced higher incidence of diarrhea, renal disorder and grade 3–4 radiation proctitis during preoperative CRT. Similarly, Arrieta et al. (2010) noted that chemotherapy-induced toxicity in non-small cell lung cancer (NSCLC) patients treated with paclitaxel and cisplatin was associated with malnutrition and hypoalbuminemia. Recently, many studies found that patients with malnutrition or weight loss ≥5 % during chemotherapy had higher risk of severe gastrointestinal toxicity, thus suggesting that evaluation of nutritional status was helpful in identifying patients at higher risk of severe gastrointestinal toxicity (Arrieta et al. 2015; Sanchez-Lara et al. 2013). It is worthy to note that nutritional changes during neoadjuvant treatment should be taken into consideration when adjusting chemotherapy and radiotherapy doses, in order to minimize the CRT-related toxicity in LARC patients.

Interestingly, our data revealed that overweight or obese LARC patients were more likely to experience more weight loss during preoperative CRT (shown in Table 1). Similarly, a Sweden nationwide cohort study has demonstrated that overweight patients with esophageal cancer were of a higher risk of malnutrition after esophagostomy (Martin and Lagergren 2009). The possible reason might be attributed to the higher administrated dose of chemotherapy and worse tolerability of CRT toxicity in those patients. In fact, a recent study showed that the risk of dose limiting toxicity was obviously increased in sarcopenic obese esophageal cancer patients (OR 5.54; 95 % CI 1.12–27.44, *P* = 0.04) (Anandavadivelan et al. 2016). Thus, malnutrition should be alerted during adjuvant treatment in overweight or obese LARC patients.

As we have mentioned above, BMI loss during preoperative CRT, which could compromise long-term OS, was common and occurred in 66.2 % of LARC patients. Therefore, it is necessary to pay more attention to the re-evaluation of nutritional status and nutritional balance in the process of anti-cancer therapy. Recently, a prospective study has demonstrated that nutrition support combined with treatment could prolong survival in advanced gastric cancer patients with malnutrition (median survival: 14.3 vs 9.6 months, *P* = 0.001) (Qiu et al. 2015). Another small volume randomized trial also confirmed the effectiveness of individualized nutritional counseling and early nutritional education during radiotherapy in improving long-term prognosis in colorectal cancer (Ravasco et al. 2012). Furthermore, feasible nutrition support during neoadjuvant chemotherapy helped to reduce the incidence of chemotherapy-related toxicities (Miyata et al. 2012). Thereby, nutritional intervention should be an early approach and targeted for each LARC patient who undergo preoperative CRT, including personalized dietary counseling and artificial nutrition support, based on spontaneous food intake, toxicity tolerance and nutritional status.

Several potential limitations should be acknowledged. First of all, weight loss does not totally represent malnutrition status. Other reported methods for the evaluation include descending change in serum albumin and hemoglobin level during treatment, as well as the scored Patient Generated Subjective Global Assessment (PG-SGA) (Das et al. 2014). Nevertheless, weight loss is closely related to those parameters, and our data provided an essential clue for further research. Moreover, we failed to evaluate the impact of different body composition changes during CRT on the patient’s oncologic outcome. Skeletal muscle and visceral adipose tissue loss might be the genuine factors contributing to poor survival according to the previous report (Dalal et al. 2012). Finally, 5-year survival outcome was unavailable due to insufficient follow-up time. This limitation may have led to underestimating the weight loss impact on long-term survival. Despite the limitations mentioned above, our study indeed raised the importance of emphasizing the nutritional status changes and nutrition support during neoadjuvant treatment for LARC patients.

## Conclusions

Severe weight loss during preoperative CRT compromised the survival outcome of patients with LARC. Routine accurate monitoring of weight change, patient education and nutritional counseling, as well as proper supplement for nutritional balance during neoadjuvant treatment, are suggested as the integral part of the multidisciplinary approach for treating LARC patients.
